# Subject-Level Classification of Osteonecrosis of the Femoral Head from Wearable IMU Gait Data Using Multilevel Feature Fusion

**DOI:** 10.3390/bioengineering13080922

**Published:** 2026-08-14

**Authors:** Xin Yu, Yan Wang, Tiancheng Ma, Xinwu Duan, Jianxiong Ma

**Affiliations:** 1 School of Biomedical Engineering and Technology, Tianjin Medical University, Tianjin 300070, China; 2 Tianjin Orthopedic Institute, Tianjin 300050, China; 3 Tianjin Institute of Integrated Traditional Chinese and Western Medicine for Orthopedics, Tianjin 300050, China; 4 Tianjin Key Laboratory of Orthopedic Biomechanics and Medical Engineering, Tianjin 300050, China

**Keywords:** osteonecrosis of the femoral head, inertial measurement unit, wearable sensors, gait classification, multilevel feature fusion, deep learning, subject-level internal validation

## Abstract

Imaging underpins the diagnosis and structural staging of osteonecrosis of the femoral head (ONFH) but does not directly quantify functional impairment during weight-bearing walking. We developed a subject-level ONFH classification framework using multilevel gait features acquired with wearable inertial measurement units (IMUs). Thirty healthy controls and 21 participants with imaging-confirmed ONFH completed self-paced walking trials recorded at 100 Hz. Gait cycles were segmented from bilateral foot-contact events, normalized to 120 points, and represented as 17-channel kinematic waveforms, 22-dimensional cycle-level scalar features, and 7-channel dynamic absolute asymmetry waveforms. These inputs were encoded by CNN–CBAM–BiLSTM, multilayer perceptron, and one-dimensional convolutional branches, respectively, and fused at the feature level. Evaluation used 51-fold leave-one-subject-out cross-validation, training-fold-only preprocessing, within-subject probability averaging, and five predefined random seeds. The five-seed ensemble achieved an accuracy of 0.9412, sensitivity of 0.8571, specificity of 1.0000, F1-score of 0.9231, and area under the receiver operating characteristic curve of 0.9556. Ablation analysis identified the scalar-feature vector as the principal source of incremental performance; the dynamic asymmetry branch contributed complementary information only in the complete model. These findings provide preliminary evidence for further evaluation of wearable gait-based ONFH classification in independent cohorts and objective functional assessment.

## 1. Introduction

Osteonecrosis of the femoral head (ONFH) is a progressive disorder initiated by disruption of the femoral head blood supply and subsequent osseous necrosis, with potential progression to subchondral fracture, femoral head collapse, and secondary joint degeneration [1]. Once subchondral fracture or collapse has occurred, both the available joint-preserving treatments and their expected efficacy become limited. Detecting disease and monitoring functional status before substantial structural deterioration are therefore clinically important [1,2]. Magnetic resonance imaging enables early lesion detection, whereas the Association Research Circulation Osseous (ARCO) classification stratifies structural severity [1,3,4]. Neither approach, however, directly quantifies functional impairment during weight-bearing walking. Objective and reproducible measures of dynamic function are consequently needed to complement imaging-based structural assessment.

Gait analysis provides quantitative measures of movement restriction and compensatory behavior during weight-bearing walking. Recent studies of ONFH have documented alterations in hip, knee, and ankle ranges of motion; interjoint coordination; spatiotemporal gait characteristics; and bilateral symmetry [5,6]. Optical motion capture provides detailed three-dimensional kinematic and kinetic data but depends on dedicated laboratory space, marker placement, and standardized procedures [7]. Wearable IMUs are portable, require less specialized infrastructure, and facilitate repeated assessment. Recent systematic reviews support their use for measuring spatiotemporal gait parameters and joint kinematics, while also showing that sensor placement, calibration, and algorithmic variation affect measurement consistency [8,9]. Validation studies of the Noraxon myoMOTION system have reported good agreement for sagittal-plane lower-limb kinematic waveforms; non-sagittal-plane measurements and absolute joint angles nevertheless require more cautious interpretation [10]. Studies of other walking tasks similarly suggest that sagittal-plane lower-limb kinematics are generally the most robust, although error profiles vary by joint, plane, and population [11]. Wearable systems have also been applied to pathological gait assessment in real-world settings and to classification of gait patterns associated with orthopedic disorders [12,13]. Collectively, this evidence supports IMU-based gait assessment, provided that sensor placement, calibration, and acquisition procedures are rigorously standardized.

Computational gait analysis commonly uses continuous time-series representations, handcrafted features, or hybrid approaches [14,15,16]. Continuous waveforms retain local morphology and temporal dependencies throughout the gait cycle; however, when the number of independent participants is limited, the model must learn local variation, long-range structure, and interindividual heterogeneity from sparse data. Low-dimensional descriptors are more readily interpretable but inevitably discard some dynamic information. Multilevel representations offer a means of integrating these complementary information sources, although model stability, interpretability, and external validation remain major limitations in clinical gait machine learning [17,18]. Phase-dependent analysis based on predefined gait intervals can help localize model sensitivity across the gait cycle [19]. A further methodological concern is that each participant contributes multiple correlated gait cycles. Random cycle-level splitting can place data from the same participant in both training and test sets, leading to information leakage and optimistic performance estimates [20]. TRIPOD+AI and PROBAST+AI therefore emphasize explicit reporting of the unit of analysis, data partitioning, performance assessment, and intended context of use [21,22].

Accordingly, we developed and internally evaluated a wearable gait-analysis framework for subject-level ONFH classification. The framework integrates kinematic waveforms, cycle-level scalar features, and dynamic absolute asymmetry waveforms to represent local temporal patterns, global cycle characteristics, and bilateral differences. Each representation was processed by a dedicated encoder matched to its dimensional and temporal structure before feature-level fusion, rather than being directly concatenated at the raw-input level. Training-fold-only preprocessing, subject-level leave-one-subject-out cross-validation, and two-stage probability aggregation were used to align model development with the intended subject-level prediction target. Waveform encoder comparisons, input ablation, cross-seed stability analysis, and channel-by-time-interval occlusion were performed to examine architectural choices, incremental input contributions, stochastic training variability, and the input regions most influential to model predictions.

## 2. Materials and Methods

### 2.1. Participants and Ethical Approval

This study was conducted at the Tianjin Key Laboratory of Orthopedic Biomechanics and Medical Engineering. The cohort comprised 51 participants: 30 healthy controls and 21 participants with ONFH. The primary analysis was binary classification between healthy controls and participants with ONFH. The study protocol was reviewed and approved by the Medical Ethics Committee of Tianjin Hospital, Tianjin, China (approval code: 2026 Medical Ethics Review No. 101; approved 8 May 2026). Written informed consent for participation in the study was obtained from all participants before data collection.

Participants were eligible if they met the following criteria: (1) age ≥ 18 years; (2) ONFH confirmed by magnetic resonance imaging (MRI) or computed tomography (CT) for the ONFH group, or no history of ONFH or other condition materially affecting lower-limb motor function for the healthy control group; (3) ability to stand and walk independently without an assistive device or assistance from another person; and (4) ability to understand and complete the prescribed walking task at a self-selected usual pace. Exclusion criteria were (1) a neurological disorder with the potential to affect motor control or gait; (2) a musculoskeletal disorder other than ONFH, lower-limb injury, or previous lower-limb surgery that materially affected gait; and (3) severe cardiovascular or respiratory disease, or any other chronic condition that could compromise walking safety.

For the ONFH group, lesion laterality and ARCO stage were extracted from clinical imaging records, and hip function was assessed using the Harris Hip Score. For participants with bilateral involvement, the reported ARCO stage corresponded to the clinically predominant hip. These clinical variables were used only for descriptive characterization and were not incorporated into model development or subgroup analyses.

Age, sex, height, and body mass were recorded for all participants, and body mass index (BMI) was calculated. These variables were not included as inputs to the primary gait-classification model but were subsequently evaluated in additional demographic benchmark and age-adjusted sensitivity analyses.

### 2.2. Wearable Gait Acquisition and Cycle Preprocessing

**Figure 1:**
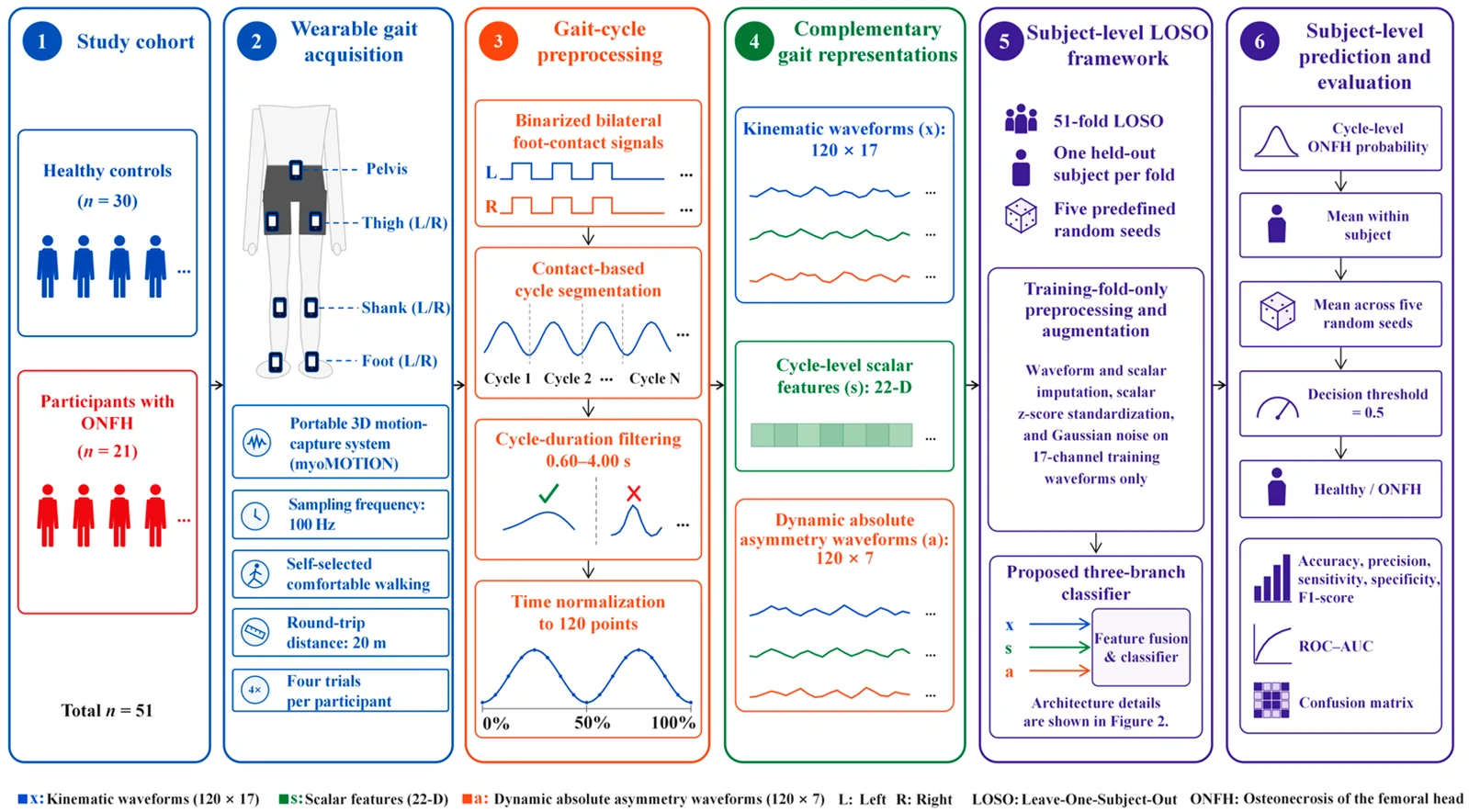
Overall study workflow. The pipeline comprised wearable gait acquisition, gait-cycle preprocessing, construction of complementary gait representations, subject-level LOSO training, and probability aggregation. x: 17-channel kinematic waveforms; s: 22-dimensional cycle-level scalar features; a: 7-channel dynamic absolute asymmetry waveforms. Ellipses indicate additional participants, gait cycles, and signal or feature elements omitted from the schematic for visual clarity.

Gait data were acquired using a portable three-dimensional motion-capture system (myoMOTION, Noraxon U.S.A., Inc., Scottsdale, AZ, USA). Seven inertial sensors were secured to the pelvis, bilateral thighs, bilateral shanks, and bilateral feet, and signals were sampled at 100 Hz. Participants completed four standardized 20-m out-and-back walking trials at a self-selected comfortable speed without using an assistive device or receiving physical assistance from another person. Each trial began from quiet standing. Participants walked 10 m along a straight walkway, performed a U-turn toward the same predefined side using a self-selected turning strategy, returned 10 m along the same path, and came to a natural stop after reaching the starting position. No dedicated acceleration or deceleration zones were prescribed. Before testing, sensor placement was verified using a standardized procedure, and the system was calibrated with the participant in the prescribed initial posture to reduce variability related to sensor positioning and initial alignment. Figure 1 summarizes the acquisition and analysis workflow.

Continuous recordings from the entire out-and-back trial were processed. Foot-contact signals were first binarized, and transient state segments shorter than three samples were removed. Rising edges of left and right foot contact were then identified, and a complete gait cycle was defined as the interval between successive ipsilateral foot-contact events. Only complete cycles lasting 0.60–4.00 s were retained to exclude clearly incomplete or incorrectly segmented intervals. No position- or task-phase-based exclusion was applied. Consequently, complete cycles satisfying the duration criterion were eligible for analysis irrespective of their location within the standardized out-and-back trial, whereas incomplete signal segments before the first and after the last detectable complete cycle were not included. Retained cycles were linearly interpolated to 120 normalized time points. Foot-contact signals were used exclusively for cycle segmentation and calculation of contact-phase features and were not included as raw waveform inputs. Within each LOSO fold, all distribution-dependent imputation and standardization parameters were estimated from the training participants only and then applied unchanged to the held-out participant.

### 2.3. Construction of Multilevel Gait Features

#### 2.3.1. Continuous Kinematic Waveforms

The first input, x, had dimensions of 120 × 17 and comprised pelvis pitch, pelvis rotation, and pelvis heading; bilateral hip flexion, hip abduction, and hip external rotation; bilateral knee flexion; and bilateral ankle dorsiflexion, ankle abduction, and ankle inversion. This representation retained multijoint angular trajectories and their temporal relationships across the complete gait cycle. Missing values were imputed using channel-specific means estimated from the current training fold. The kinematic waveforms were not Z-score-standardized, thereby preserving their original angular scales and the intrinsic amplitude differences between joints.

#### 2.3.2. Cycle-Level Scalar Features

The second input, s, was a 22-dimensional vector of cycle-level biomechanical scalar features: cycle duration and the original number of samples per cycle (2 features); the bilateral mean and left–right absolute difference in range of motion (ROM) for seven paired joint variables (14 features); and six contact-phase features. The foot defining the current gait cycle was treated as the reference side. Contact-phase features comprised the reference-side stance proportion, contralateral contact proportion, double-support proportion, no-support proportion, and the relative timing of the first toe-off and subsequent foot contact on the reference side. Missing values were imputed using feature-specific means estimated from the current training fold. Both training and held-out data were then standardized using the corresponding training-fold means and standard deviations.

#### 2.3.3. Dynamic Absolute Asymmetry Waveforms

The third input, a, comprised 7-channel dynamic absolute asymmetry waveforms with dimensions of 120 × 7, defined as the pointwise absolute left–right differences $\left|L\left(t\right)-R\left(t\right)\right|$ across the normalized gait cycle. The seven channels represented hip flexion, hip abduction, hip external rotation, knee flexion, ankle dorsiflexion, ankle abduction, and ankle inversion. This representation encoded neither left–right directionality nor the clinically affected side and was therefore intended to complement, rather than replace, the original bilateral kinematic waveforms.

### 2.4. Three-Branch Feature Fusion and Consistency Optimization

**Figure 2:**
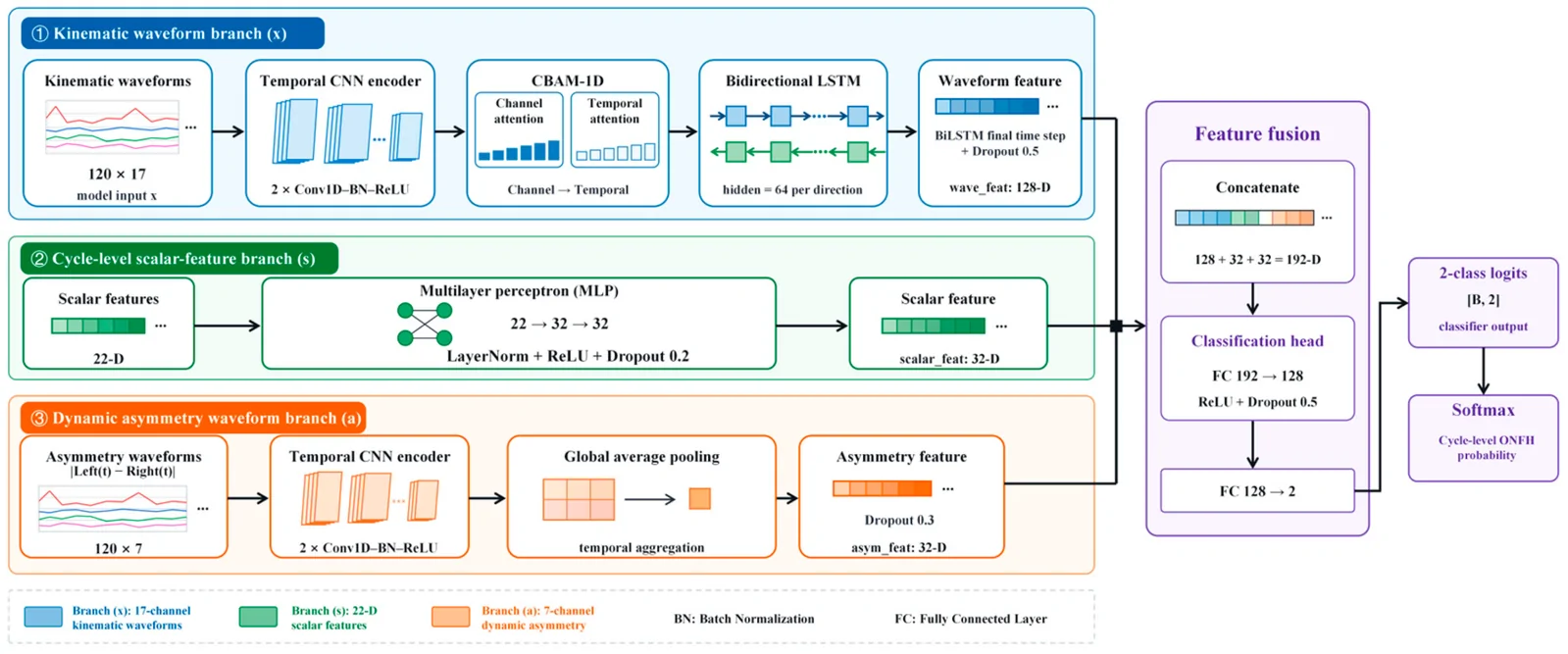
Three-branch feature-fusion model. Kinematic waveforms were encoded by a temporal CNN, CBAM-1D, and bidirectional LSTM; cycle-level scalar features were encoded by an MLP; and dynamic absolute asymmetry waveforms were encoded by a temporal CNN and global average pooling. The fused representation was mapped to two-class logits and converted into a cycle-level ONFH probability using softmax. Ellipses indicate additional waveform channels, intermediate network elements, or feature-vector components omitted from the schematic for visual clarity.

The model comprised three parallel branches (Figure 2). In the kinematic waveform branch, two one-dimensional convolutional layers with batch normalization and rectified linear unit (ReLU) activation extracted local temporal patterns. A one-dimensional convolutional block attention module (CBAM-1D) then performed adaptive channel and temporal recalibration. A single-layer bidirectional long short-term memory network (BiLSTM; 64 hidden units per direction) subsequently modeled bidirectional temporal dependencies and generated a 128-dimensional waveform representation. The cycle-level scalar-feature branch used a 22 → 32 → 32 multilayer perceptron (MLP) with layer normalization, ReLU activation, and dropout to produce a 32-dimensional representation. The dynamic asymmetry waveform branch used two one-dimensional convolutional layers and global temporal average pooling to generate a further 32-dimensional representation. The three outputs were concatenated into a 192-dimensional vector and passed through a 192 → 128 → 2 classification head to generate two-class logits, which were converted into cycle-level ONFH probabilities using softmax. Previous wearable-gait studies have shown that convolutional, recurrent, bidirectional, and attention-based architectures can learn gait events and temporal dependencies from IMU signals [23]. LSTM and bidirectional recurrent networks provide established mechanisms for long-range dependency modeling and bidirectional contextual encoding, respectively [24,25]. Attention mechanisms can adaptively weight informative channels or time points [26], and the combination of CBAM with convolutional–recurrent architectures may strengthen discriminative representations in binary time-series classification [27].

To jointly address class imbalance, within-class dispersion in the learned representation, and within-participant variability in cycle-level predictions, the total objective function was defined in Equation (1):

(1) $$L=L_{\mathrm{CB}-\mathrm{Focal}}+0.005L_{\mathrm{center}}+0.005L_{\mathrm{consistency}}$$

Class-balanced focal loss extends standard focal loss by weighting each class according to its effective number of samples in the current training fold. The unnormalized weight assigned to class c was defined in Equation (2):

(2) $$w_{c,\mathrm{raw}}=\frac{1-\beta }{1-\beta^{n_{c}}}$$

$n_{c}$ denotes the number of samples from class c in the current training fold, γ=2.0, and β=0.9999. Class weights were subsequently normalized to sum to 2, thereby maintaining a consistent overall loss scale. Center loss was used to encourage compact within-class representations in the embedding space. In combination, these terms reduce the dominance of readily classified samples, rebalance class contributions, and constrain within-class dispersion [28,29]. For each participant represented by at least two cycles in a training batch, consistency loss was calculated as the mean squared deviation of the cycle-level ONFH probabilities from that participant’s batch-specific mean probability; values were then averaged across eligible participants. Optimization used AdamW, which decouples weight decay from the adaptive gradient update [30]. The learning rate was $1\times 10^{-3}$, weight decay was $1\times 10^{-4}$, batch size was 32, and all models were trained for 50 epochs. Data augmentation was restricted to the 17-channel continuous kinematic waveforms in the training fold and consisted of Gaussian noise with a standard-deviation ratio of 0.01. Model architecture and hyperparameters were fixed before the final five-seed evaluation and were applied consistently across all LOSO folds. No setting was selected or adjusted using the held-out participant in any fold. Test data were never augmented.

### 2.5. Subject-Level Validation and Ensemble Inference

Model performance was assessed using 51-fold leave-one-subject-out cross-validation (LOSO). In each fold, all gait cycles from one participant formed the test set, while data from the remaining 50 participants were used for training; thus, no participant contributed data to both sets. Parameters for waveform imputation, scalar-feature imputation, and scalar-feature standardization were estimated solely from the current training fold. To characterize variability arising from random initialization and stochastic optimization, the complete pipeline was repeated independently using random seeds 5, 42, 64, 777, and 1024. The five random seeds were used to characterize stochastic training variability and construct the final ensemble; they were not treated as independent validation samples.

Inference involved two levels of aggregation. Within each random-seed run, cycle-level ONFH probabilities were arithmetically averaged across all cycles from the held-out participant to obtain one subject-level probability. The five seed-specific subject-level probabilities were then averaged to obtain the final ensemble probability. The classification threshold was fixed at 0.5 and was not tuned using held-out predictions.

### 2.6. Comparative, Ablation, and Model-Interpretation Analyses

Four waveform encoders—1D-CNN, BiLSTM, CNN–BiLSTM without CBAM, and CNN–CBAM–BiLSTM—were compared while all other inputs, auxiliary branches, loss terms, and training hyperparameters were held constant. The 1D-CNN served as a controlled deep-learning baseline within the same multilevel-input framework. A linear support vector machine (SVM) using the 22 cycle-level scalar features was additionally evaluated as a conventional fixed-feature baseline under the same 51-fold subject-level LOSO protocol. Separate component ablations removed CBAM, center loss, or the subject-level consistency loss while retaining the remaining inputs, auxiliary branches, training settings, and evaluation protocol. Input ablation evaluated four configurations: the 17-channel kinematic waveforms alone; the kinematic waveforms plus the 7-channel dynamic absolute asymmetry waveforms; the kinematic waveforms plus the 22-dimensional cycle-level scalar features; and all three input representations. These analyses assessed the relative contributions of the waveform encoders and input combinations under the same five-seed subject-level LOSO protocol. Candidate waveform encoders, input configurations, and loss components were examined during preliminary model development. The final comparison set, model architecture, and training settings were then fixed before the reported five-seed LOSO evaluation and applied consistently across all folds.

Channel-by-time-interval occlusion was used to characterize model dependence on specific input regions [18]. The 17 kinematic waveform channels and 7 dynamic absolute asymmetry channels yielded 24 waveform dimensions. Based on predefined functional gait-phase boundaries, the 120-point normalized gait cycle was divided into eight intervals: initial contact (0–2%), loading response (2–12%), midstance (12–30%), terminal stance (30–50%), preswing (50–60%), initial swing (60–73%), midswing (73–87%), and terminal swing (87–100%) [19]. For each occlusion, values in the selected channel–time interval were replaced by the position-specific means estimated from the current training fold. Importance was quantified as the post-occlusion reduction in the logit margin between the true and alternative classes. Values were first averaged across cycles within each held-out participant and then aggregated with equal weighting across participants and random seeds. This procedure describes relative model sensitivity to the specified inputs; it does not identify causal disease mechanisms.

To assess the stability of the occlusion findings across stochastic training runs, the five seed-specific 24 × 8 importance maps were compared using pairwise Spearman rank correlations, Pearson correlations, and overlap among the ten highest-ranked channel–interval regions. Model complexity was summarized by the number of trainable parameters, estimated core multiply–accumulate operations and floating-point operations per gait cycle, and forward-pass latency on CPU and GPU hardware. Inference timing excluded data loading, gait-cycle segmentation, and feature preprocessing.

### 2.7. Statistical Analysis

Participant baseline and clinical characteristics were summarized descriptively. Continuous variables are presented as the mean ± standard deviation or median (interquartile range), as appropriate for their distributions, and categorical variables are presented as counts and percentages. BMI was calculated as body mass (kg) divided by height squared (m^2^). Baseline and clinical variables were used to characterize the cohort and were not involved in training, feature selection, or classification-threshold determination for the primary gait model. Age, sex, height, and BMI were additionally evaluated in the demographic sensitivity analyses described below.

Performance reporting followed the recommendation that clinical prediction-model studies present discrimination, threshold-dependent measures, and estimation uncertainty [31]. The performance measures were accuracy, precision, sensitivity, specificity, F1-score, and subject-level area under the receiver operating characteristic curve (AUC). Results from the five random-seed runs are summarized as the mean ± standard deviation to describe stochastic training variability; final ensemble measures were calculated from the ensemble probability for each of the 51 participants. Stratified participant-level bootstrapping with 5000 resamples was used to estimate 95% confidence intervals (CIs) for the F1-score and AUC [32]. Wilson 95% CIs were calculated for accuracy, precision, sensitivity, and specificity [33]. All resampling was conducted at the participant level. Given the limited number of independent participants, point estimates were interpreted together with their uncertainty intervals and cross-seed variability.

Between-model differences for the proposed model and the linear SVM, 1D-CNN baseline, model without CBAM, model without center loss, and model without subject-level consistency loss were assessed using stratified paired participant-level bootstrap resampling with 5000 resamples. Within each bootstrap sample, the same resampled participants were used for both models, and metric differences were calculated as the proposed-model estimate minus the comparator-model estimate.

Calibration of the final five-seed ensemble was evaluated at the participant level using the Brier score, a calibration plot based on five equal-frequency probability groups, and the calibration intercept and slope. Uncertainty was estimated using 5000 stratified participant-level bootstrap resamples. No post hoc recalibration or classification-threshold optimization was performed.

To assess potential demographic confounding, an age-only logistic regression model and a demographic logistic regression model including age, sex, height, and BMI were evaluated using the same 51-fold leave-one-subject-out framework as the gait model. Both models used L2 regularization with a fixed regularization parameter of C = 1.0. Within each fold, continuous predictors were standardized using the training participants only, and the classification threshold was fixed at 0.5. Differences between the gait and demographic models were assessed using stratified paired participant-level bootstrap resampling with 5000 resamples.

An exploratory Firth penalized logistic regression analysis was additionally performed using standardized age and the standardized logit-transformed out-of-fold gait probability. Profile-likelihood 95% confidence intervals and penalized likelihood-ratio tests were used for inference. This analysis was interpreted as an adjusted association analysis rather than as the separately validated performance of a combined prediction model. All data preprocessing, model development, statistical analyses, and visualization were performed in Python 3.10.20 using PyTorch 2.6.0 with CUDA 12.4, NumPy 2.2.6, pandas 2.3.3, SciPy 1.15.2, scikit-learn 1.7.2, and Matplotlib 3.10.9.

## 3. Results

### 3.1. Participant Characteristics and Dataset Composition

The analysis included 51 participants. The median age was 25 years among healthy controls and 46 years among participants with ONFH; additional baseline characteristics are reported in Table 1. Among the 21 participants with ONFH, 16 had unilateral involvement and five had bilateral involvement. The cohort included four participants with ARCO stage I, 11 with stage II, five with stage III, and one with stage IV disease. For participants with bilateral involvement, the reported ARCO stage corresponded to the clinically predominant hip. The median Harris Hip Score was 69.1 points (IQR, 63.4–76.7). All participants completed gait acquisition and provided analyzable data. After application of the duration criterion, the dataset contained 4268 complete gait cycles: 2241 from healthy controls and 2027 from participants with ONFH. The median number of retained gait cycles per participant was 79.0 (IQR, 64.3–84.0; range, 46–93) among healthy controls and 95.0 (IQR, 84.0–100.0; range, 54–189) among participants with ONFH. All performance estimates and confidence intervals treated the 51 participants—not the individual gait cycles—as the independent observational units.

In the demographic sensitivity analyses, the age-only LOSO model achieved an accuracy of 0.9020, sensitivity of 0.8095, specificity of 0.9667, F1-score of 0.8718, and AUC of 0.9810. The demographic model incorporating age, sex, height, and BMI achieved an accuracy of 0.8824, sensitivity of 0.7619, specificity of 0.9667, F1-score of 0.8421, and AUC of 0.9730. The gait model yielded higher threshold-dependent point estimates, including an accuracy of 0.9412, sensitivity of 0.8571, specificity of 1.0000, and F1-score of 0.9231. The corresponding AUC estimates were 0.9556 for the gait model, 0.9810 for the age-only model, and 0.9730 for the multivariable demographic model. Because the paired bootstrap confidence intervals included zero, the observed between-model differences should be interpreted cautiously and cannot yet be considered conclusive. In the exploratory Firth analysis, the standardized out-of-fold gait logit score remained associated with ONFH status after adjustment for age (OR = 17.92, 95% profile-likelihood CI, 1.40–1673.78; likelihood-ratio *p* = 0.016), supporting the possibility that the gait representation provides information complementary to age.

### 3.2. Subject-Level Classification Performance and Reproducibility

**Figure 3:**
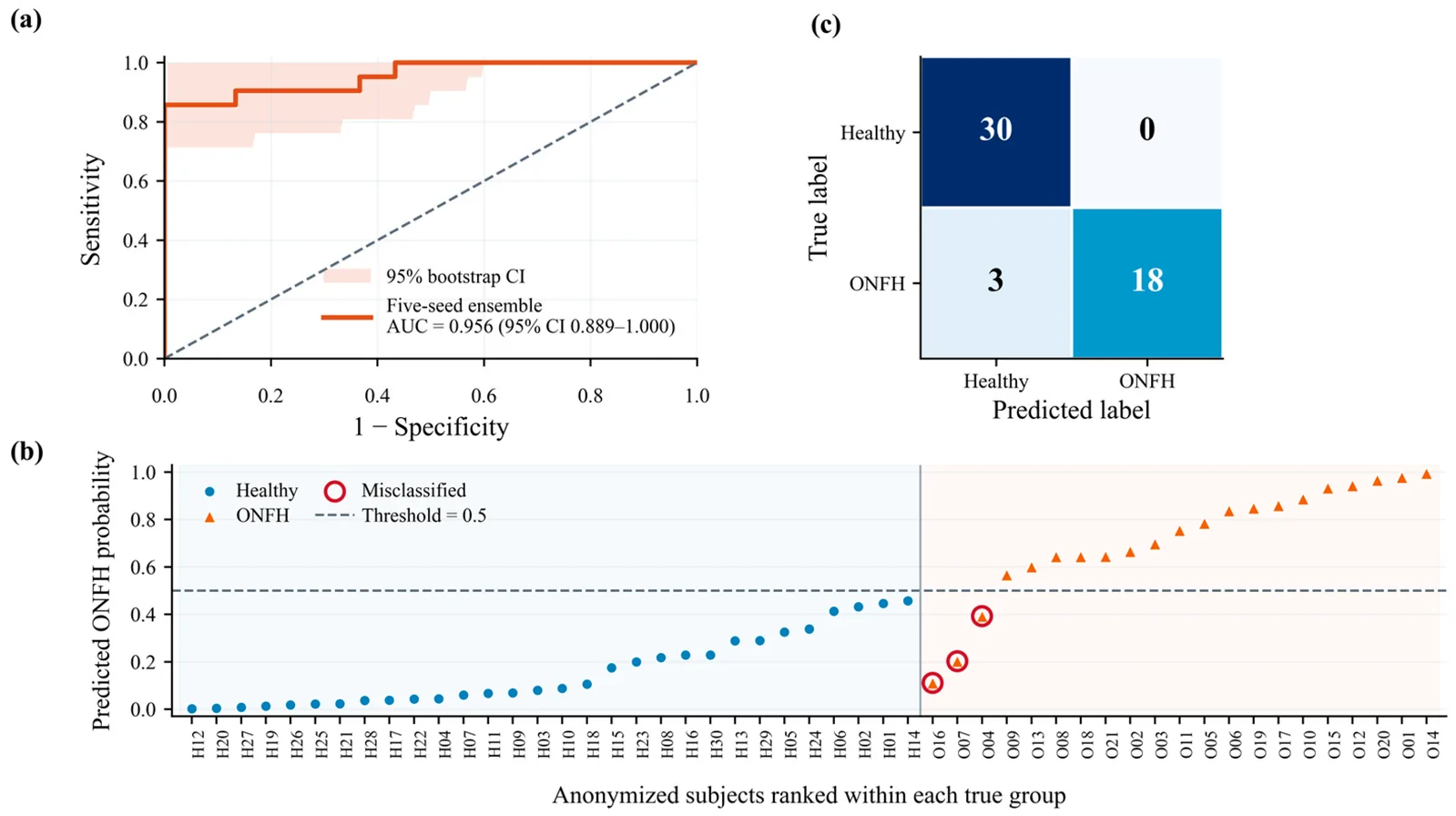
Subject-level classification performance of the five-seed ensemble. (**a**) Receiver operating characteristic curve with the 95% bootstrap confidence band; (**b**) predicted probabilities for anonymized subjects ordered within each true group, with red circles denoting misclassified subjects; and (**c**) confusion matrix. In panel (**a**), the diagonal dashed line denotes the no-discrimination reference (AUC = 0.5).

The five-seed ensemble correctly classified all 30 healthy controls and 18 of the 21 participants with ONFH, corresponding to TN = 30, FP = 0, FN = 3, and TP = 18. Subject-level accuracy, precision, sensitivity, specificity, F1-score, and AUC were 0.9412, 1.0000, 0.8571, 1.0000, 0.9231, and 0.9556, respectively (Table 2; Figure 3). Although precision and specificity both had point estimates of 1.0000, their lower 95% confidence limits were 0.8241 and 0.8865, respectively, indicating appreciable uncertainty around these estimates. Across the five individual random-seed runs, mean AUC was 0.9517 ± 0.0127; variability in the remaining measures is reported in Table 2.

### 3.3. Incremental Value of the Model Architecture and Multilevel Features

Under the common training and evaluation protocol, CNN–CBAM–BiLSTM yielded the highest mean accuracy, sensitivity, F1-score, and AUC among the four encoders evaluated (Table 3). Relative to CNN–BiLSTM, adding CBAM increased accuracy from 0.8902 to 0.9255, sensitivity from 0.7810 to 0.8476, and AUC from 0.9473 to 0.9517. Within this dataset, CBAM therefore had a larger apparent effect on threshold-dependent measures than on rank-based discrimination.

At the ensemble level, the proposed model achieved higher or equal threshold-dependent point estimates than the 1D-CNN baseline, the model without CBAM, and the model without subject-level consistency loss. Compared with the linear SVM, the proposed model yielded higher accuracy (0.9412 vs. 0.8824), sensitivity (0.8571 vs. 0.7619), specificity (1.0000 vs. 0.9667), and F1-score (0.9231 vs. 0.8421), whereas the corresponding AUC values were comparable (0.9556 vs. 0.9667). Paired participant-level bootstrap comparisons indicated that the observed between-model differences remained uncertain because the corresponding 95% confidence intervals included or touched zero. Removing center loss produced identical ensemble threshold-dependent metrics and a closely similar AUC (0.9556 vs. 0.9540). Across the five individual random-seed runs, however, the full model showed higher mean performance, suggesting that center loss contributed primarily to individual-run performance stability rather than to the final ensemble operating point (Supplementary Table S1).

The input-ablation analysis indicated unequal contributions from the three input types (Table 4). Using the 17-channel kinematic waveforms alone yielded a mean sensitivity of 0.6286. Adding the 7-channel dynamic absolute asymmetry waveforms to this representation did not improve performance. By contrast, adding the 22-dimensional cycle-level scalar features increased accuracy and AUC to 0.8980 and 0.9511, respectively, accounting for the principal input-level gain. When the dynamic asymmetry branch was subsequently added to the kinematic waveforms and scalar features, accuracy, sensitivity, and F1-score increased further, while AUC changed only marginally from 0.9511 to 0.9517. Within the present cohort, the dynamic asymmetry representation therefore appeared to function as a complementary component of the full fusion model rather than as an independently informative input.

### 3.4. Cross-Seed Stability of Individual Predictions

**Figure 4:**
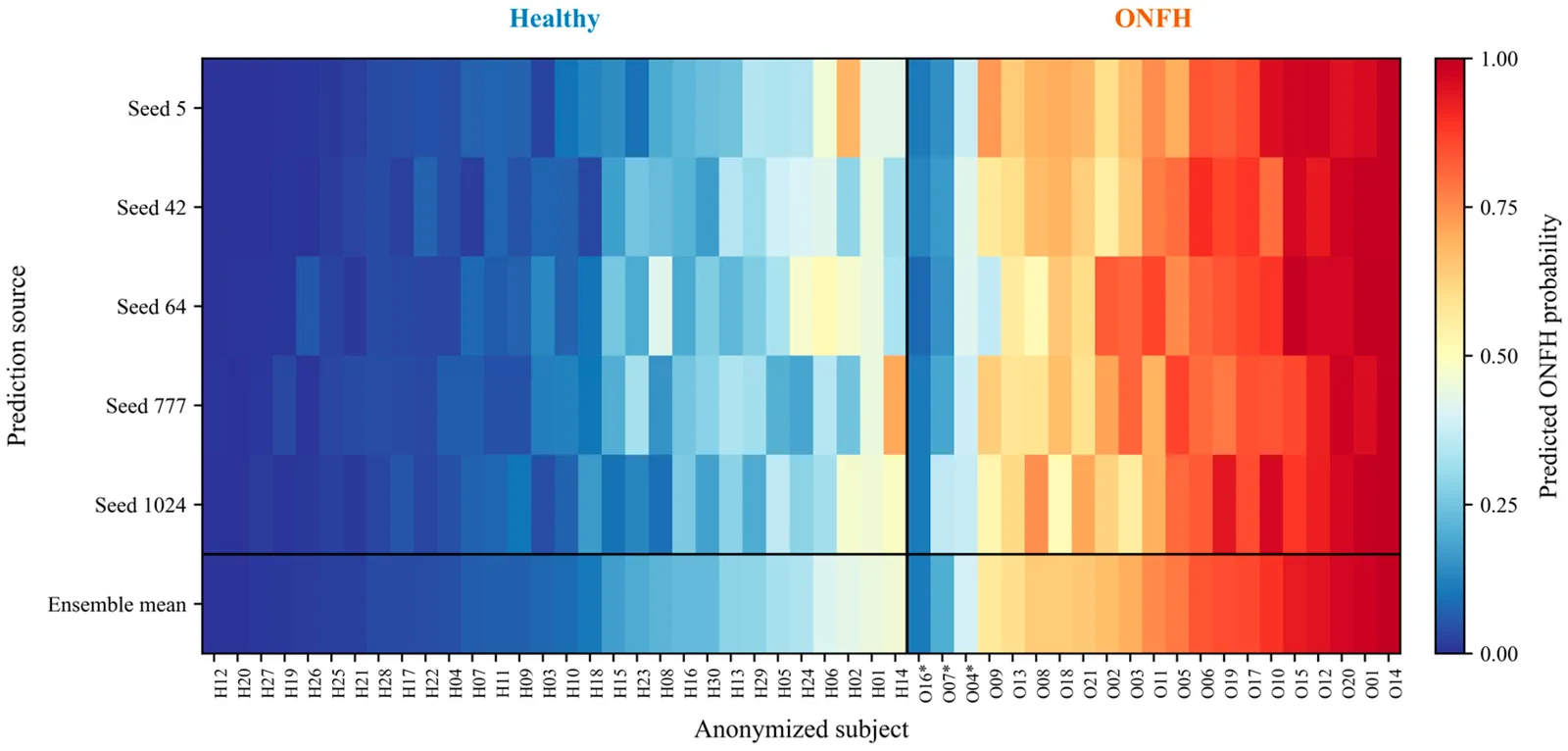
Predicted probabilities for anonymized participants under each of the five random seeds and their ensemble mean. Asterisks indicate participants with ONFH misclassified at the ensemble threshold of 0.5.

Across the five random-seed runs, most healthy participants had consistently low predicted ONFH probabilities, whereas most participants with ONFH had consistently high probabilities (Figure 4). Three participants with ONFH had probabilities near or below the classification threshold under multiple seeds, placing them close to the model’s decision boundary. The available data do not determine whether these predictions reflect disease heterogeneity, individual compensatory strategies, age-related variation, or unmeasured clinical characteristics. Future analyses incorporating more comprehensive clinical phenotyping may help characterize such borderline cases.

### 3.5. Model Dependence on Channels and Time Intervals

**Figure 5:**
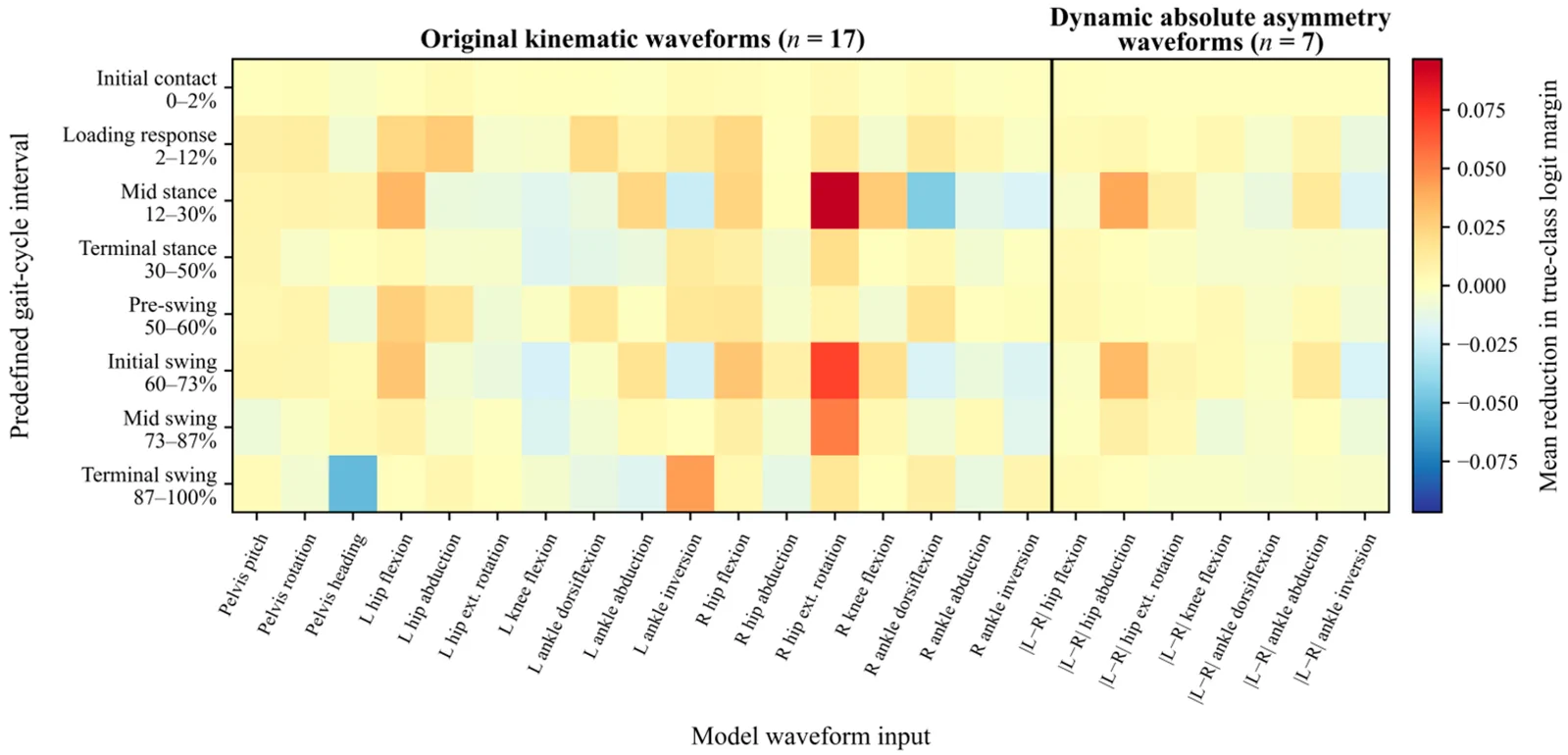
Occlusion sensitivity of 24 waveform inputs across eight predefined intervals of the normalized gait cycle. The inputs comprised 17 kinematic waveforms and 7 dynamic absolute asymmetry waveforms, calculated as pointwise absolute left–right differences. The intervals were 0–2%, 2–12%, 12–30%, 30–50%, 50–60%, 60–73%, 73–87%, and 87–100%. Values represent the mean reduction in the true-class logit margin after occlusion and were aggregated with equal weighting across participants and random seeds.

Occlusion sensitivity was concentrated in a limited number of channel–time combinations (Figure 5). The largest reductions in the true-class logit margin occurred when the right hip external-rotation waveform was occluded during the 12–30% and 60–73% intervals. Most cells had values close to zero, and several were negative. Because the intervals were defined using normalized-time boundaries rather than participant-specific physiological events, and because correlated inputs remained available during each occlusion, these findings identify model-sensitive regions only. The occlusion patterns showed moderate-to-high consistency across random seeds. Pairwise Spearman correlations across the 192 channel–interval regions ranged from 0.628 to 0.770, while Pearson correlations ranged from 0.703 to 0.838. The three highest-ranked regions—right hip external rotation during 12–30%, 60–73%, and 73–87% of the normalized gait cycle—showed positive importance and appeared among the ten highest-ranked regions in all five seed-specific analyses. Thus, the dominant right-hip external-rotation pattern was not attributable to a single random initialization. Nevertheless, the highlighted channel–interval regions should be interpreted as model-sensitivity findings rather than independent biomarkers or mechanistic evidence.

### 3.6. Calibration and Computational Profile

At the participant level, the five-seed ensemble achieved a Brier score of 0.0852 (95% CI, 0.0504–0.1268). The calibration intercept was 0.5078 (95% CI, −1.2475 to 1.5070) and the calibration slope was 1.8606 (95% CI, 1.1613–22.0452). The five-bin calibration plot showed clear separation between the lowest- and highest-probability groups, with greater variation in the intermediate probability range (Supplementary Figure S1).

The final model contained 126,960 trainable parameters, corresponding to approximately 0.484 MB of FP32 parameter storage. The estimated core computational cost was 11.82 MMACs, equivalent to 23.63 MFLOPs per gait cycle. At a batch size of one, mean forward-pass latency was 1.86 ms on the tested CPU and 1.60 ms on an NVIDIA GeForce RTX 3050 Laptop GPU. Sequential inference using the five ensemble members required 9.55 ms and 7.56 ms per cycle on the CPU and GPU, respectively.

## 4. Discussion

### 4.1. Principal Findings

This study established an end-to-end analytical pipeline from wearable gait acquisition and cycle-level representation learning to subject-level classification. Four findings warrant emphasis. First, the five-seed ensemble yielded an AUC of 0.9556 and an F1-score of 0.9231 in subject-level internal validation, with limited variation in aggregate performance across seeds. Second, comparisons with the 1D-CNN and linear SVM baselines and the component-ablation models supported the contribution of the proposed architecture primarily to fixed-threshold subject-level classification. Third, the 22-dimensional cycle-level scalar features accounted for the principal input-related performance gain, whereas the dynamic asymmetry branch contributed complementary information only in the complete fusion model. Fourth, the calibration, computational, and cross-seed occlusion analyses provided complementary evidence regarding probability quality, implementation cost, and explanation stability. Together, these results support the feasibility of combining structured biomechanical scalar features with deep temporal representations for subject-level ONFH classification.

Previous ONFH gait studies have primarily described between-group differences in joint kinematics, interjoint coordination, spatiotemporal characteristics, and bilateral symmetry [5,6]. The present study extended this work by testing whether such information could classify a participant excluded from model training. The comparatively large gain associated with the 22-dimensional cycle-level scalar features may reflect their direct encoding of low-dimensional global summaries, including cycle duration, ROM, and contact proportions. In contrast, the kinematic waveform branch must infer global cycle characteristics, local morphology, and long-range temporal dependencies from a limited number of independent participants. The ablation results therefore suggest complementarity between structured biomechanical scalar features and learned temporal representations, although this interpretation remains specific to the present model and dataset.

Building on previous advances in wearable IMU-based gait assessment and classification [12,13], the broader literature has employed diverse analytical strategies, including multimodal sensor fusion, handcrafted feature encoding, and deep temporal representation learning [14,15,16]. The present study extends this methodological foundation by integrating continuous kinematic waveforms, cycle-level scalar features, and dynamic absolute asymmetry waveforms through representation-specific encoders. Coupled with subject-level LOSO validation and within-participant probability aggregation, the proposed framework aligns multilevel gait representation learning with the intended subject-level classification objective. These methodological features extend wearable IMU-based gait analysis to ONFH and provide a coherent basis for interpreting the complementary contributions of the three input representations.

Dynamic absolute asymmetry waveforms did not improve performance when added to the kinematic waveforms alone. When combined with both the kinematic waveforms and 22-dimensional cycle-level scalar features, however, they were associated with further increases in accuracy, sensitivity, and F1-score. This representation preserves the temporal evolution of bilateral differences but encodes neither left–right directionality nor the clinically affected side. It also overlaps partially with information contained in the bilateral kinematic waveforms and ROM differences, while physiological asymmetry may occur in healthy individuals. The branch may therefore provide conditionally complementary information rather than a strong independent discriminative signal. Confirmation in independent data is needed.

### 4.2. Subject-Level Validation and Prediction Stability

Wearable gait data are inherently hierarchical because each participant contributes multiple correlated gait cycles. Randomly partitioning cycles between training and test sets can allow a model to exploit participant-specific characteristics rather than disease-associated patterns, producing information leakage and optimistic performance estimates [20]. In the present analysis, participants were treated as the independent units for LOSO partitioning, fold-specific preprocessing, bootstrapping, and final performance assessment. Cycle-level probabilities were aggregated to obtain one prediction per participant. This design aligned the analytical unit with the intended prediction target and reduced bias from treating correlated cycles as independent observations. Nevertheless, LOSO remains an internal-validation procedure, and performance should be confirmed in independent target populations.

Repeating training under five random seeds provided an empirical assessment of variability attributable to random initialization and stochastic optimization. These runs were used to characterize stochastic variability and construct the final ensemble rather than to represent independent validation cohorts. Aggregate performance varied little, and the same three ONFH cases remained close to the decision boundary across seeds. These findings suggest that the borderline predictions were not solely artifacts of a single initialization. The high observed specificity should nevertheless be interpreted in conjunction with its confidence interval.

### 4.3. Model Interpretation and Clinical Positioning

Channel-by-time-interval occlusion identified input regions to which model predictions were comparatively sensitive. The dominant right-hip external-rotation pattern remained among the highest-ranked regions across all five seed-specific analyses, indicating that this model-sensitivity finding was not driven by a single random initialization. Greater sensitivity in hip-related channels is broadly consistent with reports of altered hip motion and lower-limb interjoint coordination in ONFH [5]. Previous work has also documented changes in spatiotemporal gait characteristics and bilateral symmetry [6], suggesting that ONFH-associated gait impairment may involve multilevel alterations rather than a single joint variable. However, the intervals used here were based on fixed percentages of the normalized gait cycle and therefore did not fully correspond to physiological phases derived from each participant’s actual foot-contact and toe-off events. In addition, correlated inputs may preserve redundant information when one waveform is occluded. The analysis thus provides model-specific explanatory cues, not evidence that the highlighted regions are disease biomarkers or causal mechanisms [18]. Because the waveform channels retained their anatomical left–right orientation rather than being realigned according to the clinically predominant side, the right-sided pattern should be interpreted as model-specific spatial sensitivity rather than direct evidence of lesion laterality. The observed right-sided predominance may therefore reflect cohort- and task-specific correlations among anatomically labeled bilateral waveforms, including consistent directional characteristics of the standardized walking protocol, rather than direct encoding of the affected side; occlusion analysis alone cannot distinguish among these possibilities.

The internal calibration analysis yielded a relatively low Brier score and clear separation between the lowest- and highest-probability groups. The calibration slope estimate remained imprecise, indicating that the absolute probabilities require confirmation in independent cohorts before clinical interpretation. The compact parameter count and millisecond-scale forward-pass latency nevertheless support further evaluation of the framework in resource-conscious analytical workflows.

The proposed framework is intended to complement imaging-based structural assessment by providing quantitative information on gait function. Wearable acquisition and subject-level probability outputs may support objective functional follow-up and pre- and post-treatment comparisons after validation in independent cohorts. Future studies should confirm discrimination and calibration and evaluate threshold performance and incremental value beyond routine clinical information in relevant target populations. Their design and reporting should follow TRIPOD+AI and PROBAST+AI recommendations concerning transparency, risk of bias, and applicability [21,22].

### 4.4. Limitations and Future Directions

First, subject-level LOSO prevented participant overlap between the training and test sets, while training-fold-only preprocessing prevented information from the held-out participant from entering the estimation of imputation and standardization parameters. Repeated training under five random seeds characterized stochastic training variability. Nevertheless, this study provides internal validation in a single standardized setting with 51 independent participants. The findings therefore require confirmation in larger independent cohorts using a predefined analysis pipeline [34,35]. External studies should also examine transferability across IMU hardware, sensor-placement protocols, and nonlaboratory acquisition settings.

Second, the age distribution differed markedly between the healthy and ONFH groups and remained an important potential source of confounding. Age was not included as an input to the primary gait model; however, comfortable walking speed and other gait characteristics vary with age even among healthy adults [36]. The additional sensitivity analyses showed that age carried substantial discriminatory information in the present cohort. The gait model yielded higher threshold-dependent point estimates, whereas the demographic benchmark models yielded higher AUC estimates. Because the paired bootstrap confidence intervals included zero, these between-model differences remain uncertain and should be interpreted cautiously. Importantly, the out-of-fold gait score retained an association with ONFH status after age adjustment, supporting the possibility that wearable gait representations provide functional information complementary to age. However, the adjusted confidence interval was wide, the age distributions had limited overlap, and the cohort included only 51 independent participants. Residual age confounding therefore cannot be excluded, and validation in larger age-matched independent cohorts is required.

Third, healthy participants constituted the sole control group in this initial classification study. Inertial-sensor-based machine learning can distinguish healthy gait from gait associated with several orthopedic conditions, whereas discrimination among disorders is typically more difficult [13]. Future studies should include clinically relevant disease controls, such as patients with hip osteoarthritis, lumbar disorders, and other conditions with overlapping gait manifestations, to evaluate performance in differential-diagnosis settings. The present sensitivity analyses included demographic benchmark models based on age, sex, height, and BMI; future studies should extend these comparisons to routine clinical variables and clinically relevant disease controls.

Fourth, ARCO stage, lesion laterality, and Harris Hip Score were available for descriptive characterization but were not incorporated into model development or subgroup analyses because of the limited sample size. Disease duration, pain intensity, detailed treatment history, and side-specific disease burden were not systematically available. These factors may contribute to between-participant heterogeneity and may help explain cases near the decision boundary. Larger cohorts with comprehensive clinical phenotyping are needed to assess model performance across disease stages and clinical subgroups and to determine whether model outputs are responsive to clinically meaningful functional change.

Fifth, the standardized out-and-back protocol captured composite functional gait rather than isolated steady-state straight-line walking. Because individual gait cycles were not assigned task-phase labels, the relative contributions of straight walking, turning, and transitional gait could not be quantified separately. Future studies should incorporate task-phase annotation to compare phase-specific and composite gait representations.

Finally, channel-by-time-interval occlusion measures relative model dependence on predefined input regions and cannot establish biological mechanism [18]. Fixed normalized-time intervals also only approximate participant-specific physiological gait phases. The hip-related regions with greater occlusion sensitivity should therefore be regarded as hypotheses for further study. Although the dominant right-hip external-rotation pattern was stable across random seeds, event-aligned phase definitions, independent biomechanical measurements, and longitudinal data will be needed to determine whether these model-derived patterns generalize across independent cohorts and are clinically informative. Future studies may combine occlusion with complementary attribution approaches, such as SHAP, integrated gradients, and attention-based visualization, to further assess the reproducibility and consistency of these model-sensitive regions.

## 5. Conclusions

This study developed and internally evaluated a subject-level framework for classifying ONFH from multilevel wearable-IMU gait representations. The model integrated kinematic waveforms, cycle-level scalar features, and dynamic absolute asymmetry waveforms and used subject-level LOSO, within-participant probability aggregation, and five-seed ensembling. In the present cohort, the ensemble yielded an AUC of 0.9556 and an F1-score of 0.9231. Ablation analysis indicated that the cycle-level scalar features provided the principal incremental contribution, whereas the dynamic asymmetry branch added complementary information only in the complete model. These results provide preliminary evidence that wearable gait data contain subject-level functional patterns associated with ONFH. Independent multicenter validation with clinically relevant control groups is needed to confirm calibration, incremental value, and potential clinical utility.

## Figures and Tables

**Table 1:** Demographic and clinical characteristics and gait-cycle composition of the study cohort.

Characteristic	Healthy Controls (*n* = 30)	Participants with ONFH (*n* = 21)
Age, years	25 (25–28)	46 (36–59)
Sex, male/female, *n*	21/9	14/7
Height, cm	173.40 ± 8.33	171.52 ± 8.69
Body mass, kg	69.37 ± 12.84	72.88 ± 9.65
BMI, kg/m^2^	22.93 ± 3.10	24.77 ± 2.61
Harris Hip Score, points	—	69.1 (63.4–76.7)
Unilateral involvement, *n* (%)	—	16 (76.2)
Bilateral involvement, *n* (%)	—	5 (23.8)
ARCO stage I, *n* (%)	—	4 (19.0)
ARCO stage II, *n* (%)	—	11 (52.4)
ARCO stage III, *n* (%)	—	5 (23.8)
ARCO stage IV, *n* (%)	—	1 (4.8)
Gait cycles included in the analysis, *n*	2241	2027
Gait cycles per participant, median (IQR; range)	79.0 (64.3–84.0; 46–93)	95.0 (84.0–100.0; 54–189)

Note: Data are presented as the median (interquartile range), mean ± standard deviation, count, or count (%), as indicated; ranges are additionally provided for the number of gait cycles per participant. Clinical characteristics were available only for participants with ONFH; an em dash indicates that the variable was not applicable to healthy controls. For participants with bilateral involvement, the reported ARCO stage corresponded to the clinically predominant hip. BMI, body mass index; ARCO, Association Research Circulation Osseous. Gait-cycle counts describe the dataset composition and were not treated as independent observations for statistical inference.

**Table 2:** Subject-level classification performance and cross-seed reproducibility of the proposed model.

Metric	Five Random-Seed Runs, Mean ± SD	Five-Seed Ensemble	95% CI
Accuracy	0.9255 ± 0.0164	0.9412	0.8408–0.9798
Precision	0.9678 ± 0.0294	1.0000	0.8241–1.0000
Sensitivity	0.8476 ± 0.0213	0.8571	0.6536–0.9502
Specificity	0.9800 ± 0.0183	1.0000	0.8865–1.0000
F1-score	0.9036 ± 0.0212	0.9231	0.8333–1.0000
AUC	0.9517 ± 0.0127	0.9556	0.8890–1.0000

Note: The classification threshold was 0.5. Wilson intervals were used for accuracy, precision, sensitivity, and specificity; stratified subject-level bootstrapping was used for the F1-score and AUC.

**Table 3:** Comparison of waveform encoders using the same five random seeds and subject-level LOSO protocol.

Model	Accuracy	Precision	Sensitivity	Specificity	F1-Score	AUC
1D-CNN	0.8706 ± 0.0407	0.8835 ± 0.0573	0.7905 ± 0.0543	0.9267 ± 0.0365	0.8341 ± 0.0528	0.9394 ± 0.0176
BiLSTM	0.8784 ± 0.0377	0.9396 ± 0.0443	0.7524 ± 0.0621	0.9667 ± 0.0236	0.8352 ± 0.0540	0.9489 ± 0.0108
CNN–BiLSTM (without CBAM)	0.8902 ± 0.0107	0.9443 ± 0.0373	0.7810 ± 0.0261	0.9667 ± 0.0236	0.8542 ± 0.0135	0.9473 ± 0.0077
CNN–CBAM–BiLSTM (proposed)	0.9255 ± 0.0164	0.9678 ± 0.0294	0.8476 ± 0.0213	0.9800 ± 0.0183	0.9036 ± 0.0212	0.9517 ± 0.0127

Note: Values are the mean ± SD across five random-seed runs. Only the 17-channel waveform encoder was varied between comparisons. Paired participant-level bootstrap comparisons are reported in Supplementary Table S1.

**Table 4:** Input ablation using the same five random seeds and subject-level LOSO protocol.

Input Set	Accuracy	Precision	Sensitivity	Specificity	F1-Score	AUC
Kinematic waveforms	0.7882 ± 0.0377	0.8212 ± 0.0831	0.6286 ± 0.0621	0.9000 ± 0.0527	0.7094 ± 0.0500	0.8740 ± 0.0382
Kinematic + asymmetry	0.7843 ± 0.0367	0.8062 ± 0.0612	0.6286 ± 0.0621	0.8933 ± 0.0365	0.7053 ± 0.0533	0.8660 ± 0.0128
Kinematic + scalar	0.8980 ± 0.0215	0.9478 ± 0.0613	0.8000 ± 0.0213	0.9667 ± 0.0408	0.8665 ± 0.0237	0.9511 ± 0.0076
Full model	0.9255 ± 0.0164	0.9678 ± 0.0294	0.8476 ± 0.0213	0.9800 ± 0.0183	0.9036 ± 0.0212	0.9517 ± 0.0127

Note: Values are the mean ± SD across five random-seed runs. Kinematic, 17-channel kinematic waveforms; asymmetry, 7-channel dynamic absolute asymmetry waveforms; scalar, 22-dimensional cycle-level scalar features. The full model included all three input representations.

## Data Availability

Participant-level data are not publicly available because of privacy and ethical restrictions. Requests for access may be directed to the corresponding author and will be considered subject to the ethics approval and an appropriate data-use agreement.

## Supplementary Materials

The following supporting information can be downloaded at https://www.mdpi.com/article/10.3390/bioengineering13080922/s1, Table S1: Paired participant-level bootstrap comparisons between the proposed model and prespecified baseline and component-ablation models; Figure S1: Participant-level calibration plot of the five-seed ensemble model.
