# Supplementary material for: Subject-Level Classification of Osteonecrosis of the Femoral Head from Wearable IMU Gait Data Using Multilevel Feature Fusion
Source: Bioengineering (Basel). 2026 Aug 14;13(8):922. doi: 10.3390/bioengineering13080922 (PMC13510069; doi:10.3390/bioengineering13080922)
Supplement: Supplementary file 1 [file bioengineering-13-00922-s001.zip › Supplementary_Materials.pdf]

Supplementary Materials

for

Subject-Level Classification of Osteonecrosis of the Femoral Head from Wearable IMU Gait Data Using Multilevel Feature Fusion

Supplementary Table S1. Paired participant-level bootstrap comparisons between the proposed model and prespecified baseline and component-ablation models.

Comparator	Metric	Proposed model	Comparator estimate	Difference	95% CI
Linear SVM	Accuracy	0.9412	0.8824	+0.0588	−0.0196 to +0.1569
	Precision	1.0000	0.9412	+0.0588	0.0000 to +0.1765
	Sensitivity	0.8571	0.7619	+0.0952	−0.0952 to +0.2857
	Specificity	1.0000	0.9667	+0.0333	0.0000 to +0.1000
	F1-score	0.9231	0.8421	+0.0810	−0.0321 to +0.2132
	AUC	0.9556	0.9667	−0.0111	−0.0635 to +0.0333
1D-CNN baseline	Accuracy	0.9412	0.8824	+0.0588	0.0000 to +0.1181
	Precision	1.0000	0.8947	+0.1053	0.0000 to +0.2273
	Sensitivity	0.8571	0.8095	+0.0476	0.0000 to +0.1429
	Specificity	1.0000	0.9333	+0.0667	0.0000 to +0.1667
	F1-score	0.9231	0.8500	+0.0731	0.0000 to +0.1579
	AUC	0.9556	0.9460	+0.0095	−0.0238 to +0.0540
Model without CBAM	Accuracy	0.9412	0.9020	+0.0392	0.0000 to +0.0980
	Precision	1.0000	0.9444	+0.0556	0.0000 to +0.1667
	Sensitivity	0.8571	0.8095	+0.0476	0.0000 to +0.1429
	Specificity	1.0000	0.9667	+0.0333	0.0000 to +0.1000
	F1-score	0.9231	0.8718	+0.0513	0.0000 to +0.1324
	AUC	0.9556	0.9540	+0.0016	−0.0222 to +0.0286
Model without subject-level consistency loss	Accuracy	0.9412	0.9216	+0.0196	0.0000 to +0.0588
	Precision	1.0000	0.9474	+0.0526	0.0000 to +0.1579
	Sensitivity	0.8571	0.8571	0.0000	0.0000 to 0.0000
	Specificity	1.0000	0.9667	+0.0333	0.0000 to +0.1000
	F1-score	0.9231	0.9000	+0.0231	0.0000 to +0.0665
	AUC	0.9556	0.9524	+0.0032	−0.0127 to +0.0206
Model without center loss	Accuracy	0.9412	0.9412	0.0000	0.0000 to 0.0000
	Precision	1.0000	1.0000	0.0000	0.0000 to 0.0000
	Sensitivity	0.8571	0.8571	0.0000	0.0000 to 0.0000
	Specificity	1.0000	1.0000	0.0000	0.0000 to 0.0000
	F1-score	0.9231	0.9231	0.0000	0.0000 to 0.0000
	AUC	0.9556	0.9540	+0.0016	−0.0127 to +0.0159

Note: Differences were calculated as the proposed-model estimate minus the comparator-model estimate using 5000 stratified paired participant-level bootstrap resamples. Confidence intervals that included or touched zero indicated uncertainty in the corresponding between-model difference. The classification threshold was fixed at 0.5. CBAM, convolutional block attention module; AUC, area under the receiver operating characteristic curve.

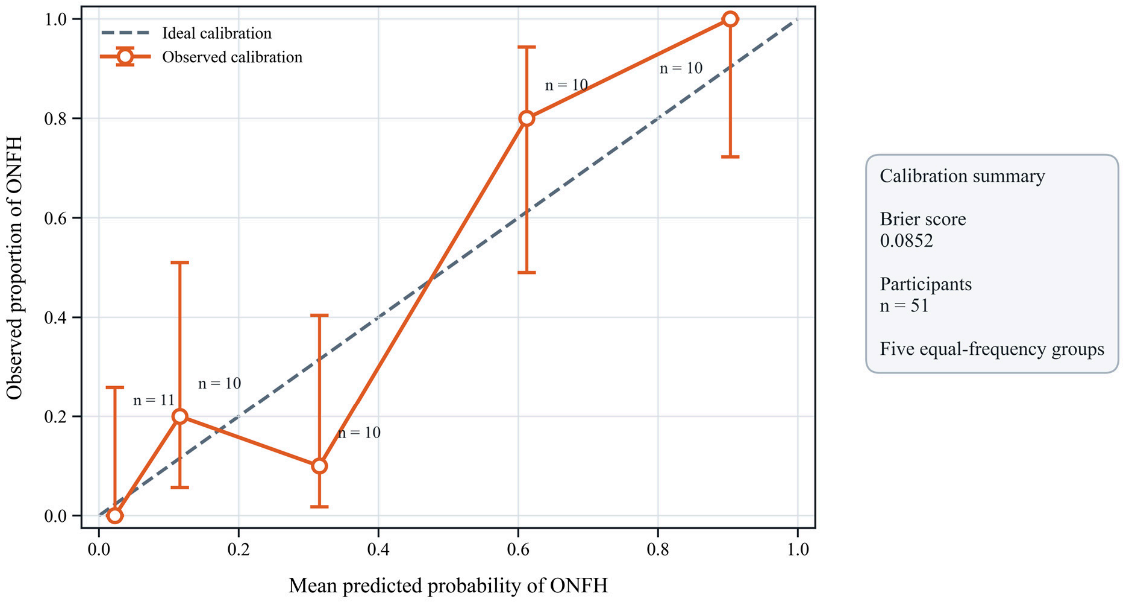

Supplementary Figure S1. Participant-level calibration plot of the five-seed ensemble model. Participants were divided into five equal-frequency groups according to the predicted probability of ONFH. Points represent the mean predicted probability and the observed ONFH proportion in each group, and error bars represent Wilson 95% confidence intervals. The dashed diagonal line indicates ideal calibration. The Brier score was 0.0852.
